# Resveratrol Inhibits Breast Cancer Stem-Like Cells and Induces Autophagy via Suppressing Wnt/β-Catenin Signaling Pathway

**DOI:** 10.1371/journal.pone.0102535

**Published:** 2014-07-28

**Authors:** Yujie Fu, Hui Chang, Xiaoli Peng, Qian Bai, Long Yi, Yong Zhou, Jundong Zhu, Mantian Mi

**Affiliations:** 1 Research Center for Nutrition and Food Safety, Third Military Medical University; Chongqing Key Laboratory of Nutrition and Food Safety, Chongqing, China; 2 Institute of Pharmacy and Bioengineering, Chongqing University of Technology, Chongqing, China; 3 Department of Public Health, School of Preclinical Medicine, Chengdu Medical College, Chengdu, China; Centro de Investigación en Medicina Aplicada (CIMA), Spain

## Abstract

Resveratrol, a natural polyphenolic compound, is abundantly found in plant foods and has been extensively studied for its anti-cancer properties. Given the important role of CSCs (Cancer Stem Cells) in breast tumorigenesis and progression, it is worth investigating the effects of resveratrol on CSCs. The article is an attempt to investigate the effects of resveratrol on breast CSCs. Resveratrol significantly inhibits the proliferation of BCSCs (breast cancer stem-like cells) isolated from MCF-7 and SUM159, and decreased the percentage of BCSCs population, consequently reduced the size and number of mammospheres in non-adherent spherical clusters. Accordingly, the injection of resveratrol (100 mg/kg/d) in NOD/SCID (nonobese diabetic/severe combined immunodeficient) mice effectively inhibited the growth of xenograft tumors and reduced BCSC population in tumor cells. After the reimplantation of primary tumor cells into the secondary mice for 30 d, all 6 control inoculations produced tumors, while tumor cells derived from resveratrol-treated mice only caused 1 tumor of 6 inoculations. Further studies by TEM (Transmission electron microscopy) analysis, GFP-LC3-II puncta formation assay and western blot for LC3-II, Beclin1 and Atg 7, showed that resveratrol induces autophagy in BCSCs. Moreover, resveratrol suppresses Wnt/β-catenin signaling pathway in BCSCs; over-expression of β-catenin by transfecting the plasmid markedly reduced resveratrol-induced cytotoxicity and autophagy in BCSCs. Our findings indicated that resveratrol inhibits BCSCs and induces autophagy via suppressing Wnt/β-catenin signaling pathway.

## Introduction

Breast cancer is the most common malignancy in women worldwide, with approximately 70,000 new cases diagnosed each year. Despite advances in the detection and treatment of breast cancer, mortality from this disease remains high. The National Cancer Institute (NCI) has recognized that prevention is a critical component in minimizing the number of individuals that are afflicted with breast cancer [1], [2].

The cancer stem cell hypothesis suggested that cancers be driven by a small subpopulation of stem cells. The CSCs possess such capacities as self-renewal and tumor generation [3]. The concept of CSCs has profound clinical implications for cancer prevention and therapeutic strategies. With the identification of cancer-initiating cells and the assay of nonobese diabetic/severe combined immunodeficient (NOD/SCID) mouse xenotransplants, this hypothesis is well established [4]. The initial discovery of breast CSCs revealed a cellular subpopulation from human breast cancer tumors characterized by the cell-surface markers ESA^+^/CD44^+^/CD24^−/low^ [5], [6]. This subpopulation of cells in tumors was highly tumorigenic [7]. As few as 200 ESA^+^/CD44^+^/CD24^−/low^ cells or 1000 CD44^+^/CD24^−/low^ cells, could produce tumors when xenotransplanted into NOD/SCID mice. While more than 50,000 unsorted cells were required to generate tumors [8]. Subsequent studies found that the aldehyde dehydrogenase (ALDH) activity could also be used to enrich for breast CSCs and normal mammary stem cells [9], [10]. The convenient isolation of CSCs has allowed the investigation of the molecular mechanisms involved in their origin, self-renewal, differentiation into cancer cells, resistance to radiation therapy and chemotherapy, and invasiveness and metastatic ability [11]. Clinical analyses of CSCs in breast tumors have found a correlation between the proportion of CSCs and poor prognosis [12]. Therefore, chemoprevention and therapy strategies that specifically target CSCs are an urgent need [13].

Because a diet rich in plant foods reduced the risks of cancer, there were more and more interest in isolating and characterizing the nutritive and non-nutritive components in fruits and vegetables for potential chemo-preventive agents [14], [15], [16]. Resveratrol (3,4′,5-trihydroxy-trans-stilbene) is a polyphenol compound which is abundantly found in plant foods including grapes, red wine, berries, and peanuts. Studies revealed a wide spectrum of pharmacological bioactivities of resveratrol, such as antioxidant, anti-inflammatory, anti-tumor and anti-atherosclerotic properties [17], [18], [19]. The anti-cancer effect of resveratrol has been shown to modulate various steps of carcinogenesis and development such as initiation, progression, and metastasis [20], [21]. However, the underlying molecular mechanism of anti-cancer effect has not been clearly defined. Given the important role of CSCs in breast tumorigenesis and development, it is worth investigating the effects of resveratrol on breast CSCs and exploring the potential mechanisms. The purpose of this work was to develop an understanding of the effects of resveratrol on breast CSCs to determine its therapeutic value in preventing or treating this disease.

## Materials and Methods

### Chemicals and reagents

Resveratrol (R5010), cholera enterotoxin, hydrocortisol and CQ (chloroquine) were purchased from Sigma-Aldrich (St. Louis, MO, USA); DMEM/F12, DMEM, Ham's F12 medium, FBS (fetal bovine serum) and BSA (bovine serum albumin) were purchased from HyClone (Beijing, China); Trizol reagent, horse serum, medium 199, antibioticantimycotic, gentamicin, insulin, Lipofectamine 2000, Opti-Mem were purchased from Invitrogen (Carlsbad, CA, USA); EFG and bFGF (Basic fibroblast growth factor) were purchased from PeproTech Inc (Rocky Hill, USA); B-27 was purchased from Gibco (Gaithersburg, MD, USA); The Cell Counting Kit (CCK-8) was purchased from Dojindo Laboratories (Kumamoto, Japan). Aldefluor assay kit and collagenase/hyaluronidase were purchased from StemCell Technologies (Vancouver, Canada); Antibodies to β-catenin was purchased from Bioworld Technology (Minneapolis, MN, USA); Antibodies to cyclin D1, Beclin1 and Atg 7 were purchased from Santa Cruz (CA, USA); Antibodies to LC3 were purchased from Cell Signaling Technology (Danvers, MA, USA). The GFP- LC3-II plasmids were kindly provided by Dr. Tamotsu Yoshimori (National Institute for Basic Biology, Okazaki, Japan). The plasmid of pcDNA3-S33Y β-catenin (Plasmid 19286) was provided by Addgene (MA, USA).

### Cell lines and culture

Human normal breast epithelial cell line MCF10A and breast cancer cell lines MCF-7, SUM159 were all purchased from Institute of Biochemistry and Cell Biology, Chinese Academy of Sciences (Shanghai, China). MCF10A cells were maintained in DMEM/F12 medium supplemented with mitogenic additives including 100 ng/ml cholera enterotoxin, 10 µg/ml insulin, 0.5 µg/ml hydrocortisol, 20 ng/ml EFG, and 5% horse serum. MCF-7 cells were maintained in DMEM medium supplemented with 10% FBS. SUM159 cells were maintained in Ham's F12 medium supplemented with 5% FBS, 1% antibioticantimycotic, 5 µg/ml insulin, 1 µg/ml hydrocortisone, and 4 µg/ml gentamicin. Isolated BCSCs were plated in serum-free DMEM supplemented with 1% BSA, 5 µg/ml insulin, 10 ng/ml bFGF, 20 ng/ml EGF (Epidermal growth factor), 1 µg/ml hydrocortisone and B-27 in a low cell-binding dish. After mammospheres formed, BCSCs were trypsinized and evaluated for ALDH (aldehyde dehydrogenase) stem cell markers by flow cytometry and then cultured in DMEM containing 10% FBS for 24 h before treatment with resveratrol or other reagents. The cells were maintained at 37°C in a humidified incubator in an atmosphere containing 5% CO_2_.

### Cell viability assay

The CCK-8 detection kit was used to measure cell viability according to the manufacturer's instructions. Briefly, cells were seeded in a 96-well microplate at a density of 5×10^4^/ml. After 24h, cells were treated with resveratrol. Subsequently, CCK-8 solution (20 µl/well) was added and the plate was incubated at 37°C for 2 h. Viable cells were counted by absorbance measurements with a monochromator microplate reader at a wavelength of 450 nm. The optical density value was reported as the percentage of cell viability in relation to the control group (set as 100%).

### Mammosphere formation assay

Single cells prepared from mechanical and enzymatic dissociation were plated in six-well ultralow attachment plates (Corning Inc., NY, USA) at a density of 500 to 1,000 cells/ml. Different concentrations of resveratrol were added to the culture. After 7 days of culture, the number of mammospheres was counted under a microscope and the photos were acquired.

### Aldefluor assay and the isolation of BCSCs

Aldefluor assay and BCSCs isolation was done using an Aldefluor assay kit according to the manufacturer's guidelines. Briefly, single cells were incubated in an Aldefluor assay buffer containing an ALDH substrate, bodipy-aminoacetaldehyde (1 µM per 1×10^6^ cells), for 40 to 50 minutes at 37°C. As a negative control, a fraction of cells from each sample was incubated under identical condition in the presence of the ALDH inhibitor diethylaminobenzaldehyde. Flow cytometry was used to measure and isolate the ALDH-positive cell population.

### Primary NOD/SCID mouse model

NOD/SCID mice were obtained from the Medical Experimental Animal Center of the Third Military Medical University [SCXK-(army)-2007-015]. They were bred and maintained in accordance with our institutional guidelines for the use of laboratory animals. Animal rooms were maintained at 25°C with 50% relative humidity and a 12-h light/12-h dark cycle. All animal procedures were approved by the Animal Ethics Committee of the Third Military Medical University. SUM159 cells (5×10^6^) mixed with Matrigel(BD Biosciences) were injected to the mammary fat pads of 5-week-old female NOD/SCID mice. Tumors were measured with a caliper and the volume was calculated using V =  (width^2^ × length)/2. Two weeks after the cell injection, mice were randomly separated into two groups: one group was injected (i.v.) with control (0.9% NaCl solution) and the other group was injected with 100 mg/kg resveratrol (dissolved in 0.9% NaCl solution) daily for 2 weeks.

### Dissociation of tumor cells

Mice were humanely euthanized and tumors were harvested. Tumor tissues were dissociated mechanically and enzymatically to obtain a single-cell suspension. Briefly, tumors were minced by scalpel and incubated in medium 199 mixed with collagenase/hyaluronidase at 37°C for 15 to 20 minutes. The tissues were further dissociated by pipette trituration and then passed through a 40- µm nylon mesh to produce a single-cell suspension.

### Secondary NOD/SCID mouse model

Living cells from the dissociated tumors were sorted out by fluorescence-activated cell sorting. Mice were implanted with tumor cells separately. Each secondary NOD/SCID mouse was inoculated with 5×10^4^ cells from control mouse tumors in one side of inguinal mammary fat pad and another 5×10^4^ cells from resveratrol-treated tumors in the contralateral mammary fat pad.

### TEM analysis

Cells were collected and fixed in 2% paraformaldehyde and 0.1% glutaraldehyde in 0.1 M sodium cacodylate for 2 h, post-fixed with 1% OsO_4_ for 1.5 h, washed, and stained for 1 h in 3% aqueous uranyl acetate. The samples were then washed again, dehydrated with graded alcohol, and embedded in Epon-Araldite resin (Canemco & Marivac, Lakefield, Quebec, Canada). Ultrathin sections were cut on an ultramicrotome (Reichert-Jung, Inc., Cambridge, UK), counterstained with 0.3% lead citrate, and examined on a transmission electron microscope (EM420; Koninklijke Philips Electronics N.V., Amsterdam, The Netherlands).

### Plasmid transfections

Cells were transfected using Lipofectamine 2000 in Opti-Mem, according to the manufacturer's protocol. The medium was replaced 8 h later and cells were collected for the next experiments 48 h post-transfection.

### GFP- LC3-II puncta formation assay

Cells were transfected with a plasmid expressing GFP- LC3-II using Lipofectamine 2000 for 24 h and then incubated for 6 h at 37°C. Following fixation, cells were immediately visualized by confocal microscopy. The number of GFP-LC3-II -positive puncta in each cell was counted. A cell including more than 20 puncta dots is considered to an autophagic cell. At least 100 cells were analysed per sample.

### Western blotting

Briefly, 30∼50 µg of protein were resolved by 15% sodium dodecyl sulfate polyacrylamide gel electrophoresis and then electroblotted onto polyvinylidene difluoride membranes for western blot analysis. Blots were probed with 1∶1,000-diluted primary antibodies overnight at 4°C, followed by the horseradish peroxidase-conjugated secondary antibodies. Then, the proteins were visualized by enhanced chemiluminescence exposure to X-ray film. Finally, the blots were scanned, and densitometric analysis was performed on the scanning images using Scion Image-Release Beta 4.02 software.

### Statistical analysis

Quantitative data are presented as means ± standard deviation (SD) of three experiments. The statistical analysis was conducted with the t-test and one-way analysis of variance using SPSS 13.0 statistical software (SPSS Inc., Chicago, IL, USA). A p-value <0.05 was considered statistically significant.

## Results

### Resveratrol inhibits BCSCs *in vitro*

**Figure 1:**
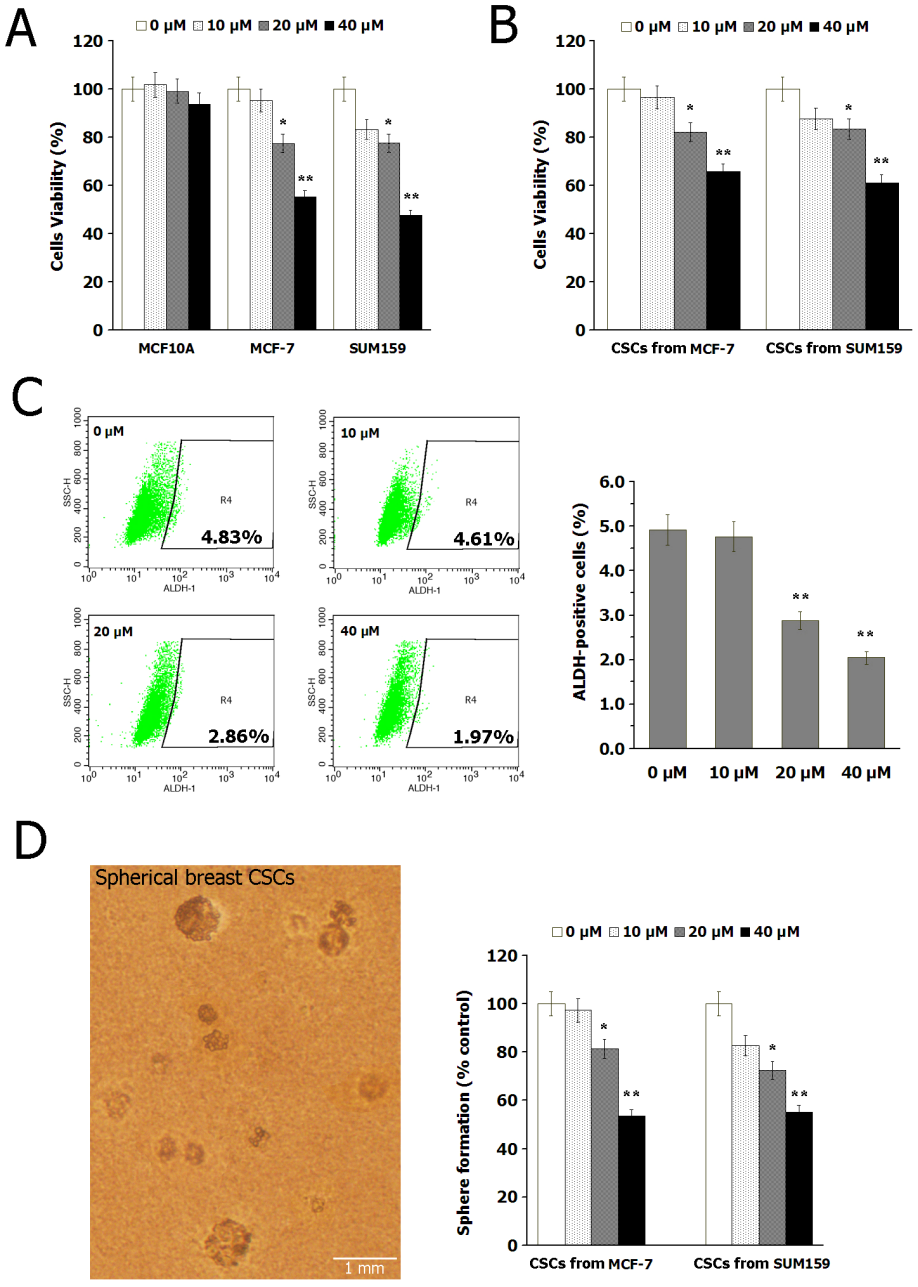
Resveratrol inhibits BCSCs *in vitro*. (A) The cytotoxic effects of resveratrol treatment (0, 10, 20 and 40 µM) for 24 h on breast epithelial cells MCF10A and breast cancer cells SUM159, MCF-7, detected by CCK-8 assay. (B) The cytotoxic effects of resveratrol treatment for 24 h on BCSCs isolated from SUM159 and MCF-7 cells. (C) Treatment with resveratrol for 24 h significantly decreased the percentage of ALDH-positive population in SUM159 cells. The changing situations in MCF-7 were alike. (D) The effect of resveratrol treatment for 7 days on the formation of mammospheres in BCSCs. Data are presented as mean ± SD (*n* = 3). ^*^ *P*<0.05, ^**^ *P*<0.01 compared with the control (0 µM).

We first evaluated the cytotoxic effects of resveratrol on breast epithelial cells MCF10A and breast cancer cells SUM159, MCF-7. Results (Fig 1A) indicated that resveratrol significantly decreased the cells viability of SUM159 and MCF-7, while showed no apparent cytotoxicity on MCF10A cells.

Then, we evaluated the inhibitory effects of resveratrol on BCSCs. As shown in Fig 1C, resveratrol treatment significantly decreased the percentage of ALDH-positive population in SUM159 cells, suggesting that resveratrol effectively reduced breast CSCs population *in vitro*. Next, cells viability assay (Fig 1B) showed that resveratrol treatment significantly inhibits BCSCs proliferation. Furthermore, we observed the effect of resveratrol on the formation of mammospheres in BCSCs, because mammary stem/progenitor cells are enriched in non-adherent spherical clusters. Results (Fig 1D) indicated that in cells treated with resveratrol compared with the control, the number of spheres declined and the size of the spheres was reduced. Taken together, these findings demonstrated that resveratrol inhibits breast CSCs *in vitro*.

### Resveratrol inhibits BCSCs *in vivo*

**Figure 2:**
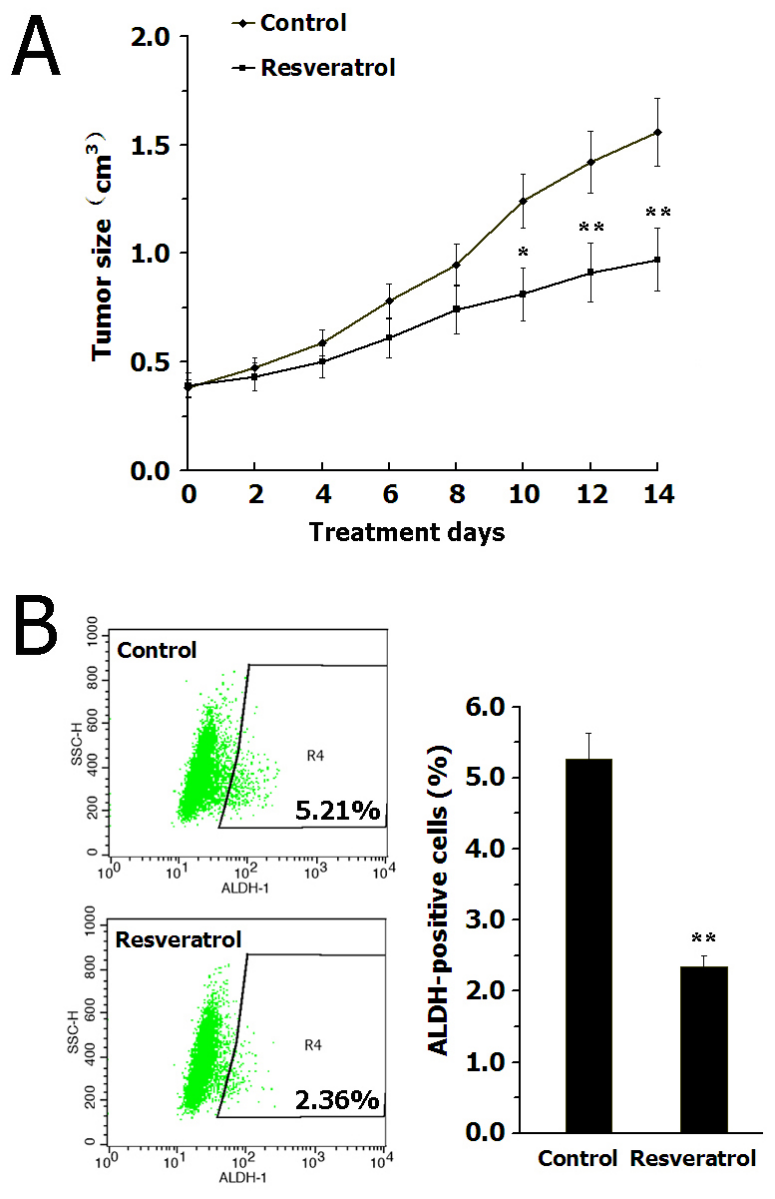
Resveratrol inhibits BCSCs *in vivo*. (A) Resveratrol injection (100 mg/kg/d, i.v.) effectively inhibits the growth of xenografted tumors of SUM159 cells in NOD/SCID mice. (B) Resveratrol administration significantly reduced the ALDH-positive populations in tumor cells dissociated from xenograft in NOD/SCID mice. Data are presented as mean ± SD (*n* = 6). ^*^ *P*<0.05, ^**^ *P*<0.01 compared with the control.

We evaluated the inhibitory effect of resveratrol targeting breast CSCs *in vivo*, using a xenograft model of SUM159 cells in NOD/SCID mice. After cell inoculation for 2 weeks, the mice were injected with resveratrol (100 mg/kg/d) or saline for another 2 weeks. In two weeks of treatment, resveratrol displayed no apparent toxicity as determined by body weight; yet the tumor volume in resveratrol-treated mice was significantly smaller than that in the control (Fig 2A), indicating that resveratrol could effectively inhibit the growth of breast cancer cells *in vivo*.

Then, the tumors were isolated from mice and the tumor cells were analyzed. The analysis revealed that resveratrol significantly reduced the ALDH-positive populations in tumor cells compared with those in the control mice (Fig 2B). Furthermore, we examined the ability of residual cancer cells to initiate tumors in NOD/SCID mice inoculated with primary tumor cells obtained from the primary xenografts. After inoculation for 30 d, all 6 control inoculations produced tumors, while tumor cells derived from resveratrol-treated mice only caused 1 tumor of 6 inoculations. These findings indicated that resveratrol could eliminate breast CSCs in primary xenografts, and consequently abrogate the regrowth of tumors in secondary mice.

### Resveratrol induces autophagy in BCSCs

**Figure 3:**
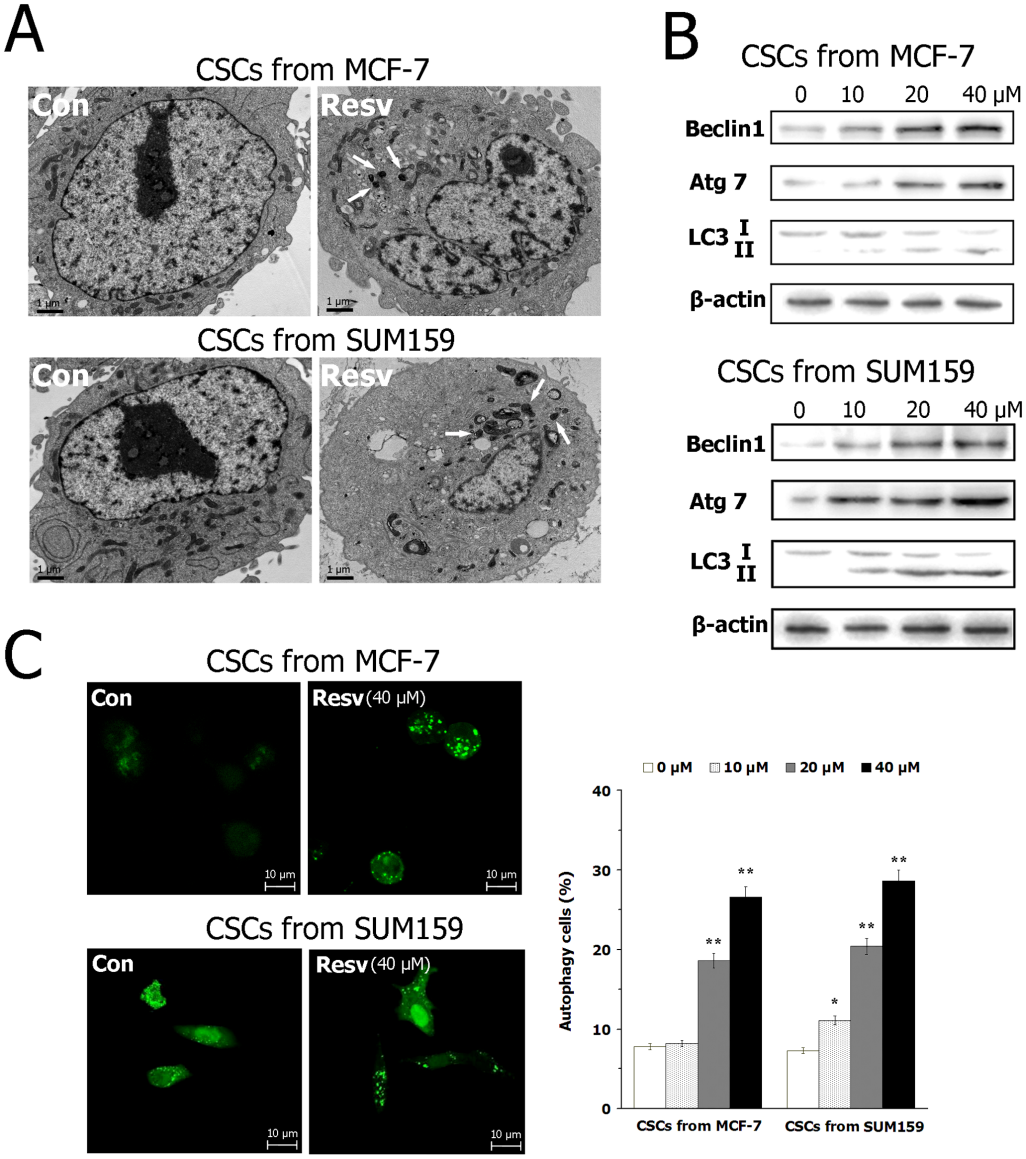
Resveratrol induces autophagy in BCSCs. (A) Resveratrol treatment (40 µM) for 24 h increased the number of autophagosomes in BCSCs from SUM159 and MCF-7, determined by TEM analysis. (B) Western blotting detection of LC3-II, Beclin1 and Atg 7 in BCSCs treated with different dose of resveratrol for 24 h. (C) GFP-LC3-II puncta formation assay in BCSCs treated with resveratrol for 24 h. Data are presented as mean ± SD (*n* = 3). ^*^ *P*<0.05, ^**^ *P*<0.01 compared with the control (0 µM).

We tested the effects of resveratrol on autophagy in breast CSCs. First, by TEM analysis, we determined that resveratrol treatment increased the number of autophagosomes in BCSCs from SUM159 and MCF-7 (Fig 3A). Then we performed western blot analysis because LC3-II, Beclin1 and Atg 7 are indicators of autophagy and they are required for autophagosome formation. As shown in Fig 3B, LC3-II, Beclin1 and Atg 7 were significantly up-regulated by resveratrol in breast CSCs in a dose-dependent manner. Furthermore, GFP-LC3-II puncta formation assay confirmed that resveratrol-treated breast CSCs displayed a significant increase in the percentage of cells with autophagosomes (GFP-LC3-II dots) compared with the control (Fig 3C). In all, these findings indicated that resveratrol induces autophagy in breast CSCs.

### Resveratrol suppresses Wnt/β-catenin signaling pathway in BCSCs

**Figure 4:**
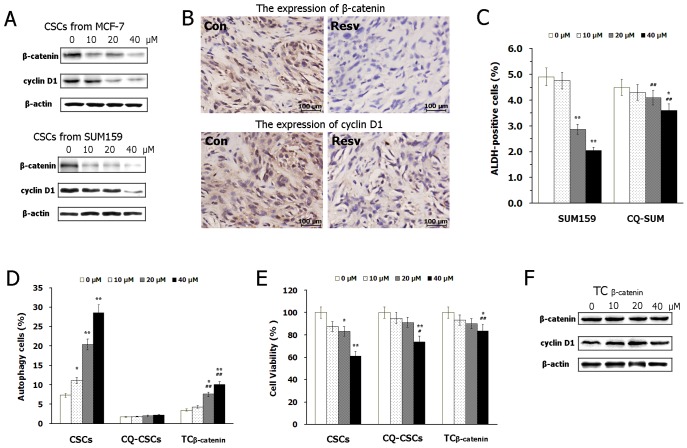
Inhibition of autophagy or over-expression of β-catenin attenuates the effects of resveratrol on BCSCs. (A) Western blot analysis of β-catenin and cyclin D1 in BCSCs treated with different dose of resveratrol for 24 h. (B) Immunohistochemistry detection of β-catenin and cyclin D1 in xenografted breast tumors. (C) The effects of resveratrol treatment for 24 h on ALDH-positive populations in SUM159 cells and SUM159 cells co-treated with 10 µM CQ (CQ-SUM). (D) The effects of resveratrol treatment for 24 h on autophagy in BCSCs, BCSCs co-treated with 10 µM CQ (CQ-CSCs) and BCSCs transfected with pcDNA3-S33Y β-catenin (TC_β-catenin_). (E) The effects of resveratrol treatment for 24 h on cells viability in BCSCs, CQ-CSCs and TC_β-catenin_ cells. (F) Western blotting analysis of β-catenin and cyclin D1 in TC_β-catenin_ cells treated with different dose of resveratrol for 24 h. Data are presented as mean ± SD (*n* = 3). ^*^ *P*<0.05, ^**^ *P*<0.01 compared with the control (0 µM); **^#^** *P*<0.05, **^##^** *P*<0.01 compared with corresponding group in CSCs.

The Wnt/β-catenin pathway is an important regulator of stem cell self-renewal, proliferation as well as autophagy. We then studied the effects of resveratrol on Wnt/β-catenin pathway in BCSCs to explore the potential anticancer mechanisms. Western blotting detections showed that resveratrol treatment decreased the expression of β-catenin and cyclin D1 in breast CSCs *in vitro* (Fig 4A). Accordingly, the immunohistochemistry detection indicated that resveratrol administration could significantly reduce the expression of β-catenin and cyclin D1 in xenograft breast tumors *in vivo* (Fig 4B).

### Inhibition of autophagy or over-expression of β-catenin attenuates the effects of resveratrol on BCSCs

We investigated the effects of autophagy inhibitor CQ on resveratrol-induced reduction of BCSCs population in SUM159 cells. As shown in Fig 4C, CQ co-treatment significantly compromises resveratrol-induced reduction of ALDH-positive populations in breast cancer cells. Then further study indicated that CQ co-treatment blocked autophagic induction of resveratrol in BCSCs, and simultaneously attenuated the anti-proliferation effect of resveratrol on BCSCs (Fig 4D and E). These data addressed the important role of autophagy in the anticancer mechanism of resveratrol on BCSCs.

To explore the role of Wnt/β-catenin pathway in the anti-proliferation and pro-autophagy effects of resveratrol, we transfected plasmid of pcDNA3-S33Y β-catenin. Transient transfection of the plasmid led to substantial production of β-catenin in BCSCs and abrogated the inhibitory effect of resveratrol on Wnt/β-catenin signal (Fig 4F). In transfected cells, over-expressed β-catenin markedly reduced resveratrol-induced cytotoxicity and autophagy, indicating that resveratrol inhibits breast CSCs and induces autophagy, at least partially, via suppressing Wnt/β-catenin signaling pathway.

## Discussion

Resveratrol is a phytoalexin that protects plants against pathogens. Specific anticancer effects of resveratrol have also been shown [22], [23], [24]. The most persuasive evidence indicates that resveratrol affects the early steps in the process of carcinogenesis [25]. The chemo-preventive as well as chemo-therapeutic effect of resveratrol against various types of cancers in pre-clinical testing has been well documented [14]. However, underlining molecular mechanism of its anti-cancer activity is yet to be defined. Increasing evidences support the CSC theory and demonstrate that CSCs have the capacity to drive tumor initiation, resistance and relapse/recurrence. Current chemopreventions and chemotherapies are not efficacious in the treatment of advanced and metastatic disease, so novel approaches are required to specifically target CSC populations. Thus, therapies that are directed against both differentiated cancer cells and CSCs may improve the treatment of these diseases. Studies have found that several dietary compounds, including curcumin and sulforaphane, are promising chemo-preventive agents against CSCs [26], [27]. Therefore, based on the chemo-preventive activity of resveratrol and the implications of the CSC theory [28], we used both *in vitro* and *in vivo* systems to determine whether resveratrol acts against breast CSCs. Our findings showed that resveratrol could significantly inhibit breast CSCs *in vitro* and *in vivo*, effectively reducing breast CSCs population, inhibiting the proliferation and the formation of mammospheres in breast CSCs. Nevertheless, it should be noted that the dose of resveratrol treatment in cellular experiments may be far higher than that in *in vivo* study; so more studies are warranted to confirm the results. Furthermore, we examined the ability of residual cancer cells to initiate tumors in NOD/SCID mice inoculated with primary tumor cells obtained from the primary xenografts. Results indicated that resveratrol administration eliminated breast CSCs in primary xenografts, and consequently abrogate the regrowth of tumors in secondary mice.

Autophagy is a catabolic process involving physiological turnover of long-lived proteins and damaged organelles in autophagosome [29]. Proper autophagy is an important response to the extrinsic and intrinsic cellular environments, and positively regulates cellular processes for survival. However, excessive autophagy will result in autophagic cell death [30], [31]. Autophagy is emerging as new target for cancer therapy and chemoprevention, yet the role of autophagy in cancer is complex, because whether autophagy causes survival or death in cancer cells is complicated and depends on specific context [32], [33]. Previous studies indicated that treatment of ovarian cancer cells and glioma cells with resveratrol and curcumin, respectively, induced autophagic cell death [34], [35]. The effect of resveratrol on autophagy in breast CSCs is unclear. Our study showed that resveratrol induces autophagy in breast CSCs, which may play an important role in the anticancer mechanism. Previous study indicated that autophagy positively regulates the CD44^+^CD24^-/low^ breast cancer stem-like phenotype [7]; however in the present study our data showed that resveratrol-induced autophagy plays a negative mechanism for BCSCs maintenance. The different observations may be attributed to that resveratrol induces excessive autophagy to cause autophagic cell death in BCSCs. More careful studies are needed to confirm the hypothesis.

CSCs have the ability to self-renew and a potential to differentiate, generating cells with a variety of phenotypes within tumors. Three signaling pathways play crucial role in the regulation of breast CSCs self-renewal, including Wnt, Notch and Hedgehog [36]. The Wnt/β-catenin signaling pathway maintains normal intestinal homeostasis and is aberrantly activated in many cancers, especially in breast cancer. β-Catenin is an essential transcriptional co-regulator in the canonical Wnt signaling pathway, forming complexes with the TCF/LEF (T cell-specific transcription factor/lymphoid enhancer-binding factor 1) family of transcription factors to control target gene expression. Recent study revealed that Wnt/β-catenin signal path also acts as a negative regulator of both basal and stress-induced autophagy [37], [38], [39]. Our study found that resveratrol suppresses Wnt/β-catenin signaling pathway in breast CSCs *in vitro* and *in vivo*. Furthermore, we explored the role of Wnt/β-catenin pathway in the anti-proliferation and pro-autophagy effects of resveratrol, results showed that over expression of β-catenin reduced autophagy rate and compromised the effects of resveratrol, indicating that resveratrol inhibits breast CSCs and induces autophagy, at least partially, via suppressing Wnt/β-catenin signaling pathway.
